# Cross-sectional survey on prevalence of attention deficit hyperactivity disorder symptoms at a tertiary care health facility in Nairobi

**DOI:** 10.1186/s13034-015-0033-z

**Published:** 2015-01-31

**Authors:** Susan Wamithi, Roseline Ochieng, Frank Njenga, Samuel Akech, William M Macharia

**Affiliations:** Department of Paediatrics and Child Health, Aga Khan University Hospital, P.O BOX 30270-00100, Nairobi, Kenya; Chiromo Lane Medical Centre, Nairobi, Kenya

**Keywords:** Paediatrics, ADHD symptoms prevalence, School performance, Injuries

## Abstract

**Background:**

Attention deficit hyperactivity disorder is the most common childhood neurobehavioral disorder with well documented adverse consequences in adolescence and adulthood, yet 60-80% of cases go undiagnosed. Routine screening is not practiced in most pediatric outpatient services and little information exists on factors associated with the condition in developing countries.

**Methods:**

This was a questionnaire based cross-sectional survey whose primary objective was to determine prevalence of attention deficit hyperactivity disorder (ADHD) symptoms in children aged 6-12 years attending a tertiary care hospital Accidents and Emergency unit. Secondary objectives were to: (i) ascertain if physical injury and poor academic performance were associated with ADHD, (ii) compare diagnostic utility of parent-filled Vanderbilt Assessment Scale (VAS) against Statistical Manual of Mental Disorders-IV (DSM-IV) as the gold reference and (iii) establish if there exists an association between ADHD symptoms cluster and co-morbid conditions.

**Results:**

Prevalence of cluster of symptoms consistent with ADHD was 6.3% (95% CI; 3.72-10.33) in 240 children studied. Those affected were more likely to repeat classes than the asymptomatic (OR 20.2; 95% CI 4.02-100.43). Additionally, 67% of the symptomatic had previously experienced burns and 37% post-traumatic open wounds. The odds of having an injury in the symptomatic was 2.9 (95% CI; 1.01-8.42) compared to the asymptomatic. Using DSM-IV as reference, VAS had a sensitivity of 66.7% (95%; CI 39.03-87.12) and specificity of 99.0% (95% CI; 96.1-99.2). Positive predictive value was 83.0% (95% CI; 50.4-97.3) and negative predictive value 98.0% (CI 95.1-99.1). Oppositional defiant disorder symptoms, anxiety, depression and conduct problems were not significantly associated with ADHD cluster of symptoms.

**Conclusion:**

The study found a relatively high prevalence of symptoms associated with ADHD. Symptomatic children experienced poor school performance. These findings support introduction of a policy on routine screening for ADHD in pediatric outpatient service. Positive history of injury and poor academic performance should trigger further evaluation for ADHD. Vanderbilt assessment scale is easier to administer than DSM-IV but has low sensitivity and high specificity that make it inappropriate for screening. It however provides a suitable alternative confirmatory test to determine who among clinically symptomatic patients requires referral to a psychiatrist.

## Background

Attention deficit hyperactivity disorder (ADHD) is the most common childhood neurobehavioral disorder [1]. Affected children experience significant adverse effects such as family conflict, injuries, academic underachievement and poor self-esteem [2]. However, 60-80% of childhood ADHD is not diagnosed and continues into adolescence and adulthood where it is associated with drug and alcohol abuse, unemployment, work and social difficulties [3]. This happens despite evidence showing that early recognition, evaluation and management of ADHD can positively redirect educational and psychosocial development of the child [4].

Few studies on ADHD have been done in Africa. Polanczyk et al. reported a worldwide pooled prevalence of ADHD of 5.3% (95% CI; 5.01-5.56) [5]. Studies done in Kinshasa and Nigeria reported ADHD prevalence of 6% and 8% respectively [6,7]. The study in Kinshasa found ADHD to be associated with both poor school performance and family health problems [6]. Atwoli et al. reported a prevalence of 9.2% in an older population of students attending a university in Kenya [8].

ADHD is associated with co-morbid conditions such as oppositional defiant disorder (ODD) in 35.2% of the affected, conduct disorders (26%), anxiety disorders (26%) and depression (18%) [9]. ADHD and co morbid conditions such as oppositional defiant disorder are associated with having more serious clinical states and poorer outcomes. In some cases ADHD is associated with antisocial behaviour and coexistence confers a worse prognosis associated with neurocognitive deficits than when the disorders are isolated. Mechanisms by which ADHD leads to antisocial behaviour remain largely unknown [10].

Childhood behavioral disorders such as ADHD, predict lower scores on academic tests and early termination of education. In a population based study of 700 children, Breslau et al. found attention problems to be the chief predictor of diminished academic achievement relative to expectations on the basis of a child’s cognitive ability. Students with inattention are inefficient learners thus limiting their acquisition of basic skills necessary for higher education [11].

Studies have shown association between ADHD and risk for unintentional injury due to behavioral risk factors such as impulsivity, inattention, risk-taking behavior and carelessness [12,13]. Medically attended injury such as head trauma or burns occurring before the age of two years may be a marker for behavioral traits of ADHD such as increased risk taking and poor impulse control [14].

Merrill et al. sought to determine if an association existed between ADHD and occurrence of injuries. Their findings were that “sprains and strains of joints, open wounds of head, neck and trunk, and upper/lower limb, and fractures of the upper/lower limb” were common. The proportion of severe injury such as “fracture of skull, neck and trunk; intracranial injury excluding those with skull fracture; and injuries to nerves and spinal cord” was three times more common in children with ADHD [15].

Diagnostic and Statistical Manual of Mental Disorders-IV (DSM-IV) is the gold standard for diagnosis of ADHD but both parents and clinicians find it complex to use [16]. This may lead to under diagnosis of ADHD. Vanderbilt Assessment Scale (VAS) was therefore developed and promoted as an alternative tool which is less complex than DSM-IV. VAS has been reworded to improve readability and is written at a third grade level [3] for easier comprehension by parents with low education. Clinicians also find it easier to administer and score the symptoms [17].

National Initiative for Children’s Healthcare Quality (NICHQ), in conjunction with the American Academy of Paediatrics (AAP) developed a tool kit for evaluation and management of children with ADHD. This toolkit is designed to be used by general practitioners and paediatricians and contains an initial evaluation form comprising of history, clinical examination and VAS for diagnosis of ADHD [18]. A similar approach was set to be evaluated in this study.

The primary purpose of this study was to determine prevalence of ADHD. The two secondary objectives were to: (i) find out whether there is association between ADHD injuries, academic performance and other co-morbid conditions (anxiety, depression, conduct disorder, or oppositional defiant disorders) and, (ii) evaluate utility of the easier to administer VAS as a screening tool for ADHD in a busy accident and emergency setting.

## Methods

The study was undertaken at the paediatric accidents and emergency (A&E) section of the Aga Khan University Hospital (AKUHN) between March and June 2012. AKUHN is a private, not for profit, tertiary health care facility based in Nairobi, Kenya. Paediatrics A&E offers a 24-hour service provided by paediatric residents and senior house officers under the supervision of paediatric registrars. Approximately 70-80 children of diverse ethnic and racial backgrounds are seen daily with an approximately equal gender distribution. Majority of children present with common acute childhood illnesses like acute respiratory tract infection, gastro-enteritis and bronchial asthma. Thus, our study population for this cross-sectional survey comprised of children with various medical and surgical conditions.

### Inclusion/Exclusion criteria

Children aged 6-12 years were enrolled provided guardians demonstrated ability to read and write in English. A written signed informed consent was also required from the primary care provider. Children on methylphenidate, antidepressants or behavioral therapy and those with neurological disorders, hearing and visual impairments or need for emergency care were excluded. Those who consented were clinically evaluated and treated for the ailments that brought them to hospital prior to completion of the self-administered study questionnaire.

Sample size was estimated at 240 based on estimated ADHD prevalence of 6% reported by Kashala et al. [5] from a neighboring country with similar socio-economic setting as Kenya.

### Ethical consideration

Study approval was obtained from the Aga Khan University Hospital Scientific and Ethical Review Committees. Enrolling of children was done after written consent from parents or primary guardians as required by the institutional review board for children under the age of 18 years. It was made clear that recruitment was entirely voluntary and that refusal to participate would not in any way compromise provision of care. Study records were secured in a locked cabinet to safeguard confidentiality.

### Data collection

Study was carried out using a two-stage ascertainment procedure. Children were evaluated for eligibility after registration at the reception between 9 am to 8 pm during week days. A maximum of 10 participants were recruited on any given day to minimize burden in the department and to hopefully capture a wider spectrum of medical conditions. Details about the study were explained to the parents by the principal investigator or the research assistant after patients had been seen by the clinician for the presenting problem. Information necessary for DSM-IV classification was obtained from parents who also completed VAS form.

Vanderbilt diagnostic parent rating scale has 55 questions divided into two sections comprising of symptoms and performance. The symptoms section contains 47 questions that are divided into various sub-sections as follows: questions 1-18 covers symptoms of ADHD, questions 19-26 oppositional defiant disorder symptoms, questions 27-40 conduct disorders and 41-47 anxiety and depression. Performance section has eight questions that indicate the level of impairment under questions 48-55. School performance, relationships with family and peers and participation in organized activities are considered under this section [18]. Hence, the tool evaluates the core symptoms of ADHD, rates the impairment ADHD may have on academic work and behavioural performance under different social settings [19].

Directions for filling out the form require parents to think about the child’s behaviour over a six month period. Additionally, the form has questions on whether patient is on medications. Symptoms scales are rated: never = 0, occasionally = 1, often = 2, very often = 3. Parent is also instructed to circle only one of the numbers on the scale. Similarly, performance scales are rated as: excellent = 1, above average = 2, average = 3, somewhat of a problem = 4, problematic = 5. The parent form contains 55 items that take approximately 10 minutes to complete [18].

Numbers for each section were tallied to meet DSM-IV criteria for diagnosis. For the predominantly inattentive subtype of ADHD, the patient was expected to score either a 2 or 3 in six out of nine questions under 1-9 and score 4 or 5 on the performance questions 48-55. To be categorized under predominantly hyperactive/impulsive subtype of ADHD, the score had to be either a 2 or 3 in six out of nine on questions 10-18 and 4 or 5 on the performance questions 48-55. ADHD combined inattention/hyperactivity required the above criteria on both inattention and hyperactivity [18].

ADHD co-morbid conditions on the form were: ODD had to score a 2 or 3 in four out of eight on questions 19-26 and score 4 or 5 on the performance questions 48-55. Conduct disorder score was 3 out of 14 on questions 27-40 and score 4 or 5 on performance questions 48-55. Anxiety/depression had to obtain 2 or 3 on three out of seven in questions 41-47 and score of 4 or 5 on performance questions 48-55 [18].

The first author (SW) or a pre-trained research assistant explained to parents how to fill out a questionnaire adapted from American Psychiatric Association, Diagnostic and statistical manual of mental disorders [16], 4th ed. Washington, D.C., 1994. SW had previously undergone training with study psychiatrist (FN) on use of the tool and subsequently trained the research assistant on its application. The following questions were inquired: (i) If their child had any of the listed symptoms of inattention that have persisted for at least six months, symptoms of hyperactivity-impulsivity that had persisted for at least six months to a degree that was inconsistent with their developmental level. The hyperactive-impulsive or inattentive symptoms that caused impairment had to have been present before age seven years. There also had to have been impairment from the symptoms in two or more settings like at school or home. Clear evidence of clinically significant impairment in social and academic functioning also had to be demonstrable [18].

Care providers of study children were requested to complete the risk assessment form with assistance provided as needed. It contained questions about school performance such as repetition of class and average end of term marks which was categorized as; below 25%, 25-50%, 50-75% or above 75%. A grade above 50% was considered as acceptable performance. Only injuries for which medical treatment was sought were considered for inclusion and categorized into burns, fractures and open wounds. Information on causes of injuries was classified under falls, fight, car accident and others. Completion of an assessment form took approximately 15 minutes after which questionnaire was scored and tabulated before providing feedback to parents. A neuro-developmental history was taken from guardians of children who screened positive for ADHD symptoms followed by a comprehensive physical examination. Visual acuity test was done using a Snellen chart and bed side testing for hearing performed using a 512 Hz tuning fork. After addressing any concerns raised by guardians, children who screened positive for ADHD were referred to a psychiatrist for re-assessment and appropriate management at a pre-negotiated subsidized cost.

The first author or research assistant explained to parents the importance of getting input from the child’s teacher. Parents were asked to consent and sign a release of information form that was then to be passed on to teachers responsible for documenting school performance feedback. Parents were asked to forward pre-stamped, self-addressed envelopes containing the DSM-IV and Vanderbilt Teacher Assessment (VTA) forms to the class teachers for completion. They were also requested to mail back completed forms to the investigator. A cover note explaining the study to teachers and instructions on how to fill the form, including a completed sample form, was enclosed in the package. The note stated that that the child would be evaluated for an undisclosed medical condition and that teachers were to complete a form on behavioural rating without specifying the actual behavioural condition. Further, they were to sign a confidentiality agreement in order to protect the privacy of the patient. Where responses delayed beyond two weeks, a telephone reminder was sent through the parents.

### Data management and analysis

Access to anonymous paper assessment forms and computerized data were limited to the principal investigator and research assistant. Data were entered in Microsoft Excel® and analysis done using STATA® Version 11 (StataCorp). Prevalence of ADHD symptoms was calculated using the number of positive cases as numerator and study population as denominator. Chi square or Fischer’s exact test were used as appropriate to compare categorical variables with P-value below 0.05 considered significant. Wilcoxon test was used for ordinal data. Odds ratios (OR) were used to determine association between ADHD symptoms and categorical variables and 95% confidence interval (CI) to determine precision around individual estimates.

## Results

A total of 240 patients between age six and twelve years were recruited over a period of four months. Their median age was nine years with an interquartile range of 7-11 years (p = 0.24). There were 15 children found to have symptoms of ADHD using DSM-IV criteria giving a prevalence of 6.3% (95% CI; 3.72-10.33). The ADHD symptomatic group was further categorized into respective subtypes as follows: hyperactive 7/15, 47.0% (95% CI; 25.21-70.13), inattentive 3/15, 20.0% (95% CI; 7.24-45.17), and combined form 5/15, 33.0% (95% CI; 15.1-58.23).

Seven children were described by parents as having inattentive (two), hyperactive (four) and combined forms (one) of ADHD symptoms. As we were unsuccessful in collecting information on social and academic function for most subjects, none was confirmed to have impairment in those aspects hence inability to determine true ADHD prevalence.

There was no sex (p = 0.89) difference between children with and without ADHD symptoms (Table 1). Age distribution was also similar in the groups (p = 0.24). A total of 72/240 (30%) children had injuries that required medical attention. Burns (63%) and open wounds (37%) were the only types of injuries reported in patients with ADHD symptoms. Symptoms were marginally associated with injuries (OR 2.86 95% CI; 0.99-8.35, p = 0.04). Both sex, (OR 1.35 95% CI; 0.82-2.32, p = 0.29), and age (OR 1.1 95% CI; 1-1.3, p = 0.14), were not significantly associated with the symptoms. Symptomatic children were as likely to score less than 50% (OR 2.17 95% CI; 0.36-12.95, p = 0.39) mark in class performance as the healthy. Similarly, there was no risk difference for scores greater than 75% (OR 0.37 95% CI; 0.12-1.15, p = 0.07). Among the few who repeated classes in the study population (8/240), those with ADHD symptoms were at a much higher risk (OR 20.2 95% CI; 4.02-100.43, p < 0.001) than those without.

**Table 1:** **Sex distribution of study patients by ADHD status**

**POPULATION**	**ADHD symptoms**	**NO ADHD symptoms**	**Total**
**Male**	8 (6.4%)	116 (93.6%)	124 (100%)
**Female**	7 (6.0%)	109 (94.0%)	116 (100%)
**Total**	15 (6.3%)	225 (93.7%)	240 (100%)

P = 0.89.

Table 2 shows diagnostic utility of the VAS in screening for ADHD using DSM-IV as the “gold-standard”. Sensitivity was 66.7% (95% CI; 39.0-87.1) and specificity 99% (95% CI; 96.1-99.2). The positive predictive value was 83.0% (95% CI; 50.4-97.3) and negative predictive value 98.0% (95% CI; 95.1-99.1). Positive likelihood ratio was 75 (95% CI; 18.3-311.2) and negative likelihood ratio 0.3 (95% CI; 0.21-0.73).

**Table 2:** **Diagnostic utility of Vanderbilt using DSM-IV gold standard**

	**DSM-IV negative**	**DSM-IV positive**	**Total**
**Vanderbilt Positive**	2	10	12
**Vanderbilt Negative**	223	5	228
**Total**	225	15	240

Sensitivity = 66.7%; Specificity = 99.0%; PPV = 88.3%; NPV = 98.0%; LR + =75; LR- = 0.3.

The study evaluated association between ADHD symptoms and co-morbidities such as oppositional defiant disorder, anxiety, depression and conduct disorders (Table 3). Only one child with ADHD symptoms suffered from anxiety (p = 0.06) while another patient had conduct disorder (p = 0.94). Respectively, six and two patients with isolated symptoms of oppositional defiant disorder were positive and negative for ADHD symptoms (p = 0.08).

**Table 3:** **Distribution of co-morbid conditions in ADHD symptomatic and Non- symptomatic children**

	**Oppositional defiant disorder**	**Conduct disorder**	**Anxiety**	**Total**
ADHD	2	1	1	4
NO ADHD	6	0	0	6
Total	8	1	1	10

## Discussion

This study found ADHD symptoms prevalence of 6.3% (95% CI; 3.72-10.33) among children visiting a busy paediatric accident and emergency unit of a tertiary care private “not-for profit” teaching hospital. Although seven children were described by parents as having ADHD (hyperactivity-impulsivity and inattention) symptoms they had no observed functional impairment hence they did not meet the diagnostic criteria for inclusion as ADHD. Further, the low response rate from teachers made it difficult to determine whether or not there was academic dysfunction attributable to the condition thus limiting our ability to estimate actual prevalence of ADHD. We unfortunately received only six reports back from teachers despite reminders. This meant we had to rely exclusively on parental reports based on observed home behavior. But parents were also expected to recall the history over the past six months which may suffer from recall bias. A combination of these factors could have contributed to underestimate of the true prevalence. The estimate is nevertheless comparable to prevalence of 5.3% and 6% in neighboring Congo and 8% in Nigeria suggesting error may be marginal [6,7]. The other studies were carried out in schools hence may not be comparable in terms of study population characteristics. Regardless of the setting, it is evident that symptoms of ADHD are prevalent enough in our population to warrant concern. In a technical review by Green et al., prevalence of ADHD in the community ranged from 4-12% compared to 2%-5% in the paediatric clinics suggesting similarity in burden in the two populations. They however observed that prevalence in paediatric clinics varied widely in the few studies available for analysis [9].

Magnitude of prevalence of ADHD is influenced by the criteria used. This type of variation is not unusual as illustrated by Wolraich et al. who encountered a similar inaccuracy in diagnosis when 4323 children were evaluated for ADHD in 10 schools in Tennessee [19]. They found a prevalence of 16% when ADHD diagnosis was based on symptoms alone compared to 6.8% when both symptoms and functional impairment was used as per diagnostic criteria requirement. In review of global prevalence of ADHD, Polanczyk et al. attributed variability to methodological differences [20]. The American Academy of Paediatrics recommends behavioral interventions for children who do not meet the full diagnostic criteria for ADHD although evidence in support of the practice is weak [16].

Unlike some other investigators, this study did not observe any gender difference between children with and without symptoms of ADHD. This could be explained by the small sample size filing to detect a true difference if it indeed existed. It could also have been caused by some unidentified seasonal occurrence like preferential referral of girls over the study period. The National Survey of Children’s Health reported a male to female prevalence ratio of 2.5:1 with clinic based populations showing 10:1 [21]. Spencer et al. attributed the gender difference to boys presenting with disruptive behaviour being referred as compared to girls with inattentive behaviour [22].

We found some association, albeit weak, between past injury, especially burns, and ADHD despite the low power of the study. Whereas a larger sample size is needed to examine this further, Tai et al. prospectively looked at “injury-proneness” of children aged six to eighteen years and found children with ADHD to have a 2-5 fold increase in risk of injury [23]. Additionally, this study found the predominant type of injury to be burns. The findings concurred with those of Fritz et al. [12].

A striking observation from our study was the up to 20-fold increase in risk of repeating classes in children with symptoms of ADHD as a manifestation of poor academic performance. This phenomenon should increase index of suspicion for ADHD among health professionals. In a study of a class of 700 by Breslau et al. on impact of early behavior disturbances on academic achievement, students with attention problems were found to be inefficient learners which limited their ability to acquire basic skills necessary for higher education [11].

Unlike other studies, ADHD in our study was not associated with oppositional defiant disorder, anxiety, depression and conduct disorders in this study [17]. This may be attributed to the fact that our study was not powered to detect such an association if it indeed exists.

VAS would clearly not be recommended for ADHD screening in view of the low sensitivity found in this study as many with the condition would be missed out. However, the high specificity and high positive likelihood ratio argue a case for its use in already suspected diagnosis from say, history of poor school performance or injury and suggestive symptoms of ADHD. Testing positive in such patients would then suggest strong need for referral to a psychiatrist for further assessment. Utility of other behavioral scales such as Conner’s Questionnaires and Strength and Difficulties Questionnaire as alternatives to DSM IV need to be further investigated in subsequent studies.

## Limitations

Among our initial intentions was to estimate prevalence of ADHD and other commonly associated behavioral conditions. We were however not successful in getting many reports back from school teachers despite reminders hence we could only determine ADHD associated symptoms without demonstrating effects on school performance and relationships with peers at school. Further, we used self- administered questionnaires rather than face-face interview that would have offered better opportunity for clarification on items that could be confusing to the respondent.

Our study was also powered to determine prevalence of ADHD symptoms but not co-morbid conditions which would call for a larger sample size. Also, as the study was conducted in a private health facility outpatient department with access limited to some members of the population, generalization should be confined to similar settings.

## Conclusions

A relatively high prevalence of ADHD symptoms in paediatric accidents and emergency departments justifies introduction of a policy on routine screening of children. Positive history of injury, especially burns, and poor academic performance should prompt clinicians to test for ADHD. Although Vanderbilt assessment scale is not adequately sensitive for use as a screening tool, it demonstrated high specificity and being easier to use in a busy service, would be an alternative to DSM-IV in determining who among the symptomatic to refer for psychiatrist assessment and management.

## Abbreviations

ADHD: Attention Deficit/Hyperactivity Disorder
DSM-IV: Diagnostic and Statistical Manual of Mental Disorders, Fourth Edition
VAS: Vanderbilt Assessment Scale
